# Cost-Effectiveness of Crisis Resolution Home Treatment for Managing Acute Psychiatric Crises in Southern Switzerland

**DOI:** 10.3389/ijph.2025.1608248

**Published:** 2025-07-14

**Authors:** Emiliano Soldini, Maddalena Alippi, Salvatore Maione, Zefiro Benedetto Mellacqua, Luca Crivelli

**Affiliations:** ^1^ Department of Business Economics, Health and Social Care, University of Applied Sciences and Arts of Southern Switzerland, Manno, Switzerland; ^2^ Cantonal Sociopsychological Organization, Ticino, Switzerland

**Keywords:** cost-effectiveness, acute psychiatric crises, crisis resolution home treatment, hospitalization, natural experiment based on geography

## Abstract

**Objectives:**

This study aimed at providing the first formal cost-effectiveness evaluation of Crisis Resolution Home Treatment (CRHT) compared to hospitalization for the management of acute psychiatric crises in Switzerland.

**Methods:**

Intervention (CRHT) and control (hospital) groups were formed based on patients’ place of residence according to a quasi-experimental design. Patients were followed starting from an acute episode of care until 2 years after discharge. Effectiveness measures were variation of psychiatric symptoms between admission and discharge and number of non-readmission days. Direct costs were obtained from the Cantonal Psychiatric Clinic and patients’ health insurance companies. Indirect costs were estimated based on sick leave certificates. Bootstrap resampling procedures and Cost-Effectiveness Acceptability Curves were used to assess cost differences between groups and cost-effectiveness.

**Results:**

CRHT resulted generally less costly than hospitalization. In the treatment phase, cost-effectiveness depended on the type of psychiatric symptoms considered, while CRHT resulted highly cost-effective in the follow-up phase.

**Conclusion:**

CRHT can be a cost-effective alternative to hospitalization for managing acute psychiatric crises in Ticino. Further research is needed to explore patients’ conditions and characteristics associated with cost-effectiveness.

## Introduction

Mental illness is one of the most frequently occurring conditions across European countries, affecting more than one out of six people and causing significant economic costs [1, 2]. Mental healthcare systems worldwide have evolved according to the socio-psychiatric approach to provide community-based alternatives to standard treatments in the psychiatric hospitals [3–5]. Crisis Resolution Home Treatment (CRHT) represents an alternative to standard inpatient treatment for managing acute psychiatric crises [6–8], increasingly adopted and recommended.

Numerous evidence in the literature showed that CRHT can be a clinically effective alternative to hospitalization for the treatment of acute psychiatric crises [7, 9–11], but evidence on cost-effectiveness of CRHT compared to standard inpatient treatment is instead rather limited. Various studies addressed the topic of CRHT economic evaluation [9, 10, 12–14]. However, many of them considered CRHT in comparison with other psychiatric community services [15–27], while some others reported evidence on the lower costs of CRHT in comparison with standard hospital treatment without a true cost-effectiveness assessment [28–31]. We found eleven studies that assessed cost-effectiveness of CRHT in comparison with inpatient treatment [12–14, 32–39]; the findings reported were mixed, with CRHT resulting sometimes more and sometimes less costly than standard hospital treatment and not always cost-effective. Moreover, the findings were hardly comparable because of the heterogeneity across studies [10]. We found important variations in terms of patients included, as, for example patients diagnosed with schizophrenia having a long treatment history [35] compared to veterans with a current psychiatric hospitalization and a high volume of previous hospitalizations [37] or to patients in the need of a hospitalization because of an acute crisis whom family is considered adherent to CRHT [14]. Moreover, we found heterogeneity regarding the type of effectiveness measures used, with, for example, the only use of the number of admission-free days during a given follow-up period [13] compared to the only use of psychiatric symptoms assessment measures [14, 37] or to a combined use of these two outcome measures [12]. Finally, the studies considered greatly differed also in terms of follow-up period length, with one study considering the treatment period only [14], and the others relying on short follow-ups up to 6 months [12, 13, 34], standard follow-ups of one [36, 39] or two [32, 37, 38] years and long follow-ups of approximately 4 years [33, 35]. The follow-up length is an important heterogeneity factor because the contributions of Knapp et al. [32, 33] showed that the clear cost-effectiveness of CRHT found after 20 months could be no longer valid after 45 months within the same study setting. There is therefore a strong need for additional detailed research on cost-effectiveness of CRHT compared to standard hospital treatment within specific homogeneous settings in terms of patients considered, effectiveness measures used and follow-up period length. Several home treatment services are nowadays active in Switzerland [40–46], with four CRHT teams representing an actual full replacement of inpatient treatment in the Cantons of Luzern [43], Aargau [44], Zürich [45] and Ticino [46]. Only three Swiss studies conducted in the Cantons of Thurgau (unpublished material), Luzern [43] and Aargau [44] addressed the issue of costs’ comparison between the CRHT and hospital settings, showing that CRHT was generally less costly even if the difference wasn’t always statistically significant. However, no cost-effectiveness assessment was carried out in any of the three studies.

The aim of our paper is to provide the first formal assessment of CRHT cost-effectiveness in comparison with standard inpatient treatment at the Swiss level. This evaluation has been designed within the unique setting of the CRHT experience in Ticino, where in 2016 an entire acute psychiatric ward was closed and replaced by a CRHT team.

## Methods

The intervention, the study design and the analysis to assess effectiveness and cost-effectiveness of CRHT in comparison with standard hospital treatment are extensively presented in the study protocol [46], the effectiveness study [11] and the synthesis working paper on cost and reimbursement of the Swiss National Science Foundation’s National Research Program “Smarter Healthcare” [47]. We provide a brief description of the intervention, while the study design, the data collected and the assessment of CRHT cost-effectiveness are presented in deeper details.

### The CRHT Intervention

In April 2016, an acute ward of the regional public psychiatric hospital (Clinica Psichiatrica Cantonale, CPC) was closed and substituted by a mobile and multidisciplinary CRHT team, available 24 h a day and 7 days a week, formed by three psychiatrists, ten mental health nurses, one team manager, one clinical psychologist and one social worker.

The treatment consisted in daily visits of about 1 hour at the patients’ home, with the possibility of multiple visits a day if necessary. Interventions were tailored according to individual patients’ needs, and included standard ingredients of acute care, such as crisis management, pharmacotherapy, psychoeducation, psychotherapy, and social care.

CRHT was available for patients aged between 18 and 65 years old with an acute psychiatric crisis for which inpatient treatment was deemed necessary by the CPC triage unit. Patients with acute intoxications and/or agitation/aggressive behaviour were excluded from the CRHT service, together with patients with a high risk of suicide/self-harm or harm to others. Also, inmates did not have access to the service. CRHT could be offered only to residents of the northern part of the Canton Ticino because of organizational and logistic reasons.

### Study Design and Sample

According to CRHT service accessibility, residents of the northern part of the Canton were included in the treatment group (CRHT) whereas patients living in the southern part formed the control group (hospital treatment) within a quasi-experimental design, more specifically a natural experiment based on geography [48]. Preliminary analyses on patients treated in the two areas of the Canton before the introduction of the CRHT service confirmed the assumption of casual distribution of relevant patients’ characteristics, showing no significant differences across geographic groups in terms of socio-demographic and clinical characteristics like gender, age, education, civil status, main psychiatric diagnosis, number of previous admissions, severity of symptoms at admission, etc. To reinforce the comparability, residents of the southern part of the Canton were included in the study only in case of willingness to accept CRHT, even if it did not imply the assignment to the treatment. Within this setting, according to the chosen design the allocation to the treatment and control groups could be considered as quasi-randomized when accounting for a set of relevant pre-treatment variables (gender, primary diagnosis, etc.).

Patients were recruited between mid-March 2017 and the beginning of April 2019. They were assessed for study eligibility according to the abovementioned CRHT inclusion and exclusion criteria. Additional inclusion criteria were the rescission of certificates of compulsory hospital detention and/or the resolution of acute drug or alcohol intoxications within 48 h from hospitalization. Additional exclusion criteria were a period of hospitalization before being transferred to CRHT exceeding 72 h and a treatment period of less than 7 days, the latter because the corresponding patients probably did not meet the criteria for an acute psychiatric crisis. Patients recruited were followed during the treatment period and a follow-up period of 2 years after discharge.

The sample size was calculated according to the main objective of the research program, which was the assessment of the clinical effectiveness of CRHT in comparison with standard hospitalization. The calculation was based on the primary endpoint, the variation in psychiatric symptoms between admission and discharge measured by the difference in the Health of the Nation Outcome Scales (HoNOS) overall score [49]. Relying on a previous study [50], according to standard statistical parameters (i.e. statistical power of 80% and 5% significance level for a bivariate test) the calculation led to a dimension of 142 patients for both groups. To assess the appropriateness of the sample size for the cost-effectiveness analysis, we performed an additional calculation considering cost differences between CRHT and standard hospital treatment found in the two Swiss studies with the most similar setting to ours [43, 44]. These highlighted a 15%–20% average cost reduction associated with CRHT; considering conservatively a 15% cost reduction hypothesis, according to the abovementioned standard statistical parameters the calculation indicated an overall sample size of 226 patients (113 per arm), which confirms the appropriateness of the initial calculation.

### Data Collected

Socio-demographic and clinical data were part of routine data; the collection of clinical data (e.g. primary psychiatric diagnosis) was performed by trained medical personnel. Cost data for the treatment phase were obtained from the CPC accounting office, while for the follow-up phase they were provided by patients’ health insurance companies.

We collected sociodemographic (gender, age, nationality, educational level, civil status, living arrangement and working conditions) and clinical (primary psychiatric diagnosis, presence of a secondary diagnosis, compulsory admission, number of previous hospitalizations and psychiatric symptoms level at admission measured by the HoNOS) data to account for differences between the intervention and control groups when comparing costs and effectiveness and evaluating cost-effectiveness.

Actual direct costs for the treatment period (central services, personnel, and operating costs) were obtained from the CPC, while reimbursed costs for the follow-up period were provided by patient’s health insurances. We adjusted the bills related to hospital treatments during the follow-up phase to account for the 55% share covered by the cantonal authorities. For both phases, indirect costs of lost production were estimated based on the number of sick leave days reported on medical certificates according to regional gender- and age-specific median gross wages.

We gathered data for two effectiveness measures. We collected the HoNOS score at admission and discharge to compute the variation in psychiatric symptoms at the end of the treatment phase, and we gathered information to calculate the number of non-readmission days (i.e. days spent outside the psychiatric hospital) for the follow-up phase.

### Statistical and Cost-Effectiveness Analyses

To account for the expected right skewness of the distributions (in particular of costs’ distributions), we used bootstrapped resampling procedures based on 5,000 repetitions to estimate both the group-specific average values of costs and effectiveness measures and the differences between the intervention and control groups. Unadjusted differences in mean costs and effectiveness measures were assessed using a bootstrapped t-test, while adjusted differences (accounting for sociodemographic and clinical characteristics) were estimated using a bootstrapped clustered regression model. Four types of confidence interval – normal-based (N), percentile (P), bias-corrected (BC), bias-corrected and accelerated (BCa) – were computed and compared to provide the results with additional robustness.

We evaluated the cost-effectiveness of CRHT compared to hospitalization based on the approach proposed by McCrone et al. [51], that we adapted to the characteristics of our setting. Cost-effectiveness was assessed according to the following net benefit (NB) function:

$${NB}_{i}=\lambda \cdot E_{i}-{TC}_{i}$$

where *E*_*i*_ is the effectiveness and *TC*_*i*_ the treatment costs. We calculated the *NB*_*i*_ for all patients in the treatment and follow-up settings with different costs and effectiveness measures:

• *Treatment setting: E*_*1*_ = HoNOS variation at discharge vs. *TC_1_* = total actual treatment costs,
• *Follow-up setting: E*_*2*_ = Number of non-readmission days during the follow-up vs. *TC_2_* = total reimbursed follow-up costs.

The theoretical societal value attributed to an increase of one unit in the effectiveness (i.e. a reduction of one point in the HoNOS at the end of the treatment or an additional day outside the psychiatric hospital during the follow-up) corresponds to the parameter λ, which is unknown. The range and the increments of λ were set separately for the treatment and follow-up phases based on NB thresholds (i.e. the values that, on average, separate negative from positive NBs). The adjusted average NB difference between the intervention and control groups was then determined using a bootstrapped clustered regression model with 5,000 repetitions accounting for the abovementioned sociodemographic and clinical characteristics, with the total treatment costs replacing the HoNOS score at admission in the follow-up analysis. CRHT cost-effectiveness probability was estimated according to the proportion of positive regression coefficients, out of the 5′000 coefficients generated for each model, for the treatment binary variable (i.e. 1 = CRHT and 0 = hospital treatment). Cost-effectiveness acceptability curves (CEAC) were finally developed based on these probabilities.

## Results

### Sample

We recruited 324 patients, 87 of which were excluded because they had a treatment length of less than 7 days or were moved to another healthcare facility before ending the treatment. We had therefore data on 237 patients for the analysis (93 in the intervention group and 144 in the control group). In the treatment phase, the adjusted difference in total actual treatment costs was calculated on 219 patients having the HoNOS total scores at admission, while the adjusted difference in the variation of the HoNOS total score between admission and discharge was calculated on 208 patients having both scores. Consequently, the cost-effectiveness assessment for the treatment phase was performed on 208 patients. The follow-up phase analysis was based on health insurance data that were available for 163 patients (75 in the intervention group and 88 in the control group).

Socio-demographic and clinical characteristics, as well as differences between intervention and control groups, were very similar for patients considered for the treatment phase and those included in the follow-up phase analysis. Overall, patients were evenly split between men and women, with a median age of 44 years old. Three-quarters were Swiss, and the educational level was equally distributed between primary and secondary (with a few patients having a tertiary level). Approximately 30% of the patients were married, 45% lived alone and slightly more than 20% were employed. From the clinical point of view, the most frequent primary diagnoses (approximately 30% each) were affective disorders (F3) and schizophrenia (F2), and approximately 60% of the patients had a secondary diagnosis. Around 25% of admissions were compulsory, and the median number of previous hospital admission was 1. The median HoNOS score (i.e. the severity of psychiatric symptoms) at admission equalled 16 points.

Table 1 – reported from the article on the study regarding the effectiveness of CRHT in comparison with inpatient treatment in Southern Switzerland [11], – presents the baseline sociodemographic and clinical characteristics of the patients considered by arm. Compared to the control group, the intervention group counted significantly more women (61.3% vs. 38.2%; p-value = 0.001) and married patients (36.6% vs. 22.2%; p-value = 0.016), as well as a lower proportion of patients living alone (34.4% vs. 51.4%; p-value = 0.010). Mental and behavioural disorders due to the use of psychoactive substances, F1 diagnosis, were less frequent in the treatment group (4.3% vs. 13.2%; p-value = 0.024), while personality and behaviour disorders in adult persons, F6 diagnosis, resulted more frequent (25.8% vs. 9.7%; p-value = 0.001). The treatment group was characterized by a significantly lower proportion of compulsory admissions rescinded before recruitment (16.1% vs. 29.9%; p-value = 0.016) and median number of previous hospitalizations at the clinic (1 vs. 2, p-value = 0.003).

**TABLE 1:** Sociodemographic and clinical characteristics of intervention and control groups (Mendrisio, Switzerland. 2019).

Characteristics	Intervention group (n = 93)	Control group (n = 144)	Statistical test for the difference^a^
Sociodemographic			
Female gender, n (%)	57 (61.3)	55 (38.2)	χ^2^ (1) = 12.173***
Age, years: median (IQR^b^)	41.7 (20.3)	45.7 (20.1)	z = 1.432
Swiss citizenship, n (%)	74 (79.6)	103 (71.5)	χ^2^ (1) = 1.969
Educational level, n (%)			
None/compulsory	40 (43.0)	67 (46.5)	χ^2^ (2) = 3.467
Secondary	47 (50.5)	59 (41.0)	
Tertiary	6 (6.5)	18 (12.5)	
Married, n (%)	34 (36.6)	32 (22.2)	χ^2^ (1) = 5.700*
Living alone, n (%)	32 (34.4)	74 (51.4)	χ^2^ (1) = 6.665*
Employed, n (%)	18 (19.4)	33 (22.9)	χ^2^ (1) = 0.429
Clinical			
Compulsory admission, n (%)	15 (16.1)	43 (29.9)	χ^2^ (1) = 6.001*
Primary diagnosis (ICD-10), n (%)			
Mental and behavioural disorders	4 (4.3)	19 (13.2)	χ^2^ (5) = 20.706**
Due to use of psychoactive substances (F1)			
Schizophrenia, schizotypal and delusional	24 (25.8)	45 (31.3)	
Disorders (F2)			
Mood [affective] disorders (F3)	29 (31.2)	41 (28.5)	
Neurotic, stress-related and somatoform	12 (12.9)	19 (13.2)	
Disorders (F4)			
Disorders of personality and behaviour in	24 (25.8)	14 (9.7)	
Adult persons (F6)			
Other disorders (F5, F8, F9, Z)	0 (0.0)	6 (4.2)	
Presence of a secondary diagnosis, n (%)	59 (63.4)	87 (60.4)	χ^2^ (1) = 0.219
Num. previous hospitalizations: median (IQR)	1 (3)	2 (4)	z = 2.887**
HoNOS at admission^c^: median (IQR)	18 (8)	16 (9)	z = −1.045

*p < 0.05, **p < 0.01, ***p < 0.001.

^a^ χ2 test for categorical variables; Fisher’s exact test was used in presence of cells with a count lower than 5. Mann-Whitney test for continuous variables; the 95% confidence intervals for p-values used to assess statistical significance were estimated using the Monte Carlo method based on 10′000 samples.

^b^ IQR, interquartile range.

^c^ Data on the HoNOS, were missing for 6 patients in the intervention group and 12 in the control group.

### Cost Data Management and Distributions’ Shape

For the treatment phase, the CPC provided us with the yearly total actual costs for each treatment arm. To calculate the average daily cost of each treatment (CRHT and hospitalization), we divided the arm-specific yearly cost by the yearly number of CRHT or hospital days. Patients’ direct treatment costs were then computed by multiplying the number of his/her number of treatment days by the arm-specific average daily treatment costs.

During the follow-up phase, seven of the patients, for which we disposed of health insurance reimbursement data, changed health insurance company, three departed out of Switzerland and three passed away. Consequently, some of the patients included in the follow-up analysis did not have a full follow-up period of 2 years; the minimum follow-up period was approximately 2 months long (66 days). To avoid the exclusion of these patients, we decided to base the analysis on the average monthly follow-up costs instead of the total costs over 2 years.

**FIGURE 1:**
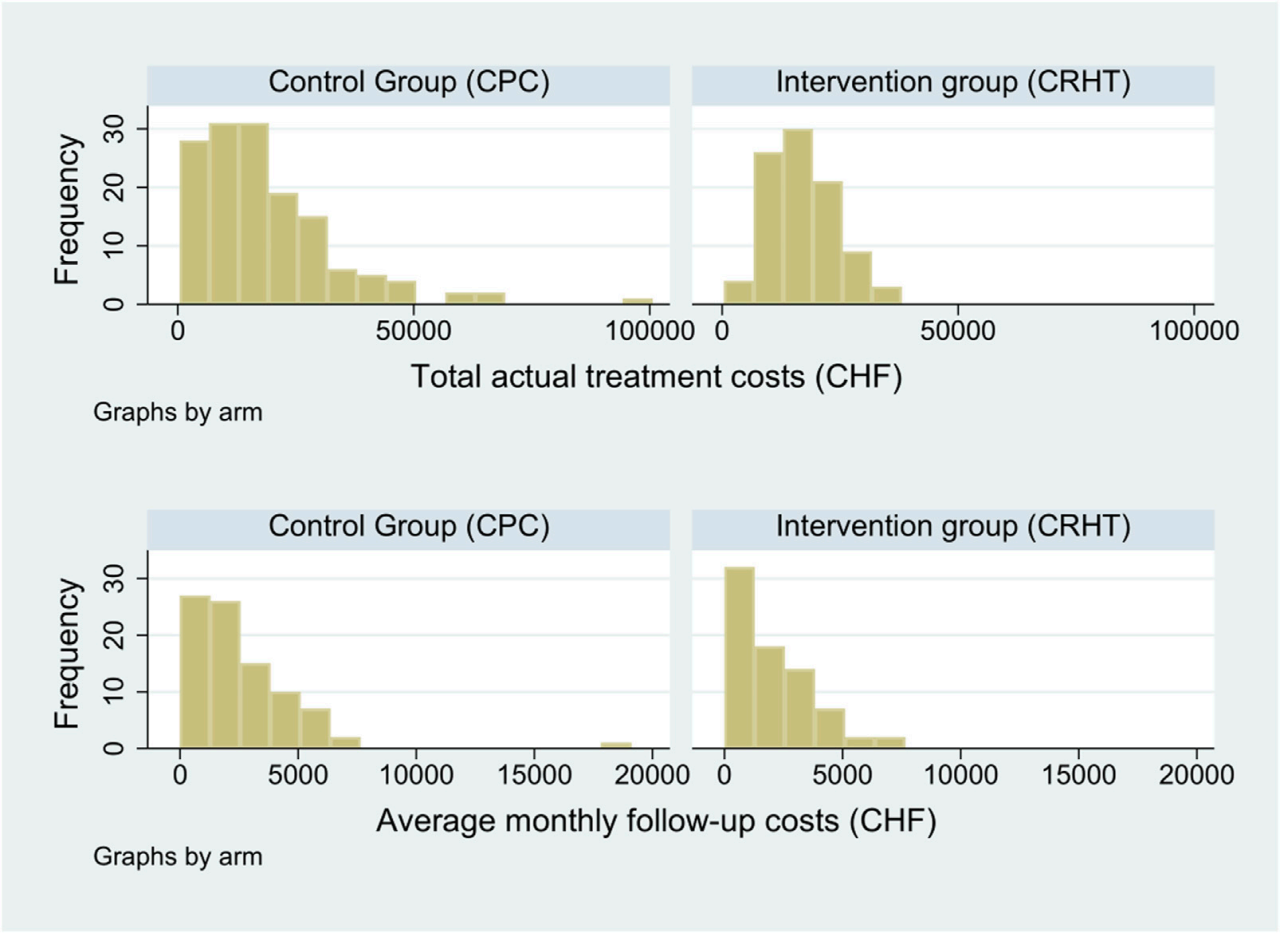
Distribution of total actual costs for the treatment phase and of average monthly reimbursed costs for the follow-up phase (Mendrisio, Switzerland. 2019).

Figure 1 illustrates the distributions of total actual costs for the treatment phase and of average monthly reimbursed costs for the follow-up phase, both including estimated costs of lost production. The right skewness of the distributions of costs, particularly emphasized for hospital treatment, justifies the bootstrap approach.

### Treatment Phase Analysis’ Results

The data reported in Table 2 indicate that CRHT unadjusted average total costs (CHF 17,064.39) were lower than those reported for hospitalization (CHF 18,888.57), even if not significantly (p-value = 0.209). The unadjusted average reduction of psychiatric symptoms between admission and discharge resulted higher for hospital treatment (−9.68 HoNOS points) compared to CRHT (−8.53 HoNOS points), but again the difference was not statistically significant (p-value = 0.216).

**TABLE 2:** Unadjusted differences in mean costs and effectiveness measures (Mendrisio, Switzerland. 2019).

Variable	Intervention group [CRHT^a^] (treatment phase: n = 93) (Follow-up phase: n = 75)		Control group [CPC^b^] (treatment phase: n = 144) (Follow-up phase: n = 88)		Bootstrap t-test for the mean difference
	Mean (bootstrap S.E.^c^)	95% confidence intervals	Mean (bootstrap S.E.)	95% confidence intervals	
Total actual costs (CHF^d^) *Treatment phase*	17,064.39	(15,607.15; 18,521.64) [N^e^]	18,888.57	(16,497.91; 21,279.24) [N]	t = 1.26
	(743.50)	(15,688.35; 18,550.18) [P^f^]	(1,219.75)	(16,505.12; 21,253.36) [P]	
		(15,679.09; 18,547.09) [BC^g^]		(16,675.13; 21,548.26) [BC]	
		(15,707.03; 18,572.46) [BCa^h^]		(16,754.85; 21,757.04) [BCa]	
Difference in the HoNOS^i^ total score between admission and discharge *Treatment phase*	−8.53	(−9.82; −7.24) [N]	−9.68	(−10.98; −8.39) [N]	t = −1.24
	(0.66)	(−9.78; −7.29) [P]	(0.66)	(−11.02; −8.38) [P]	
		(−9.79; −7.31) [BC]		(−11.02; −8.40) [BC]	
		(−9.79; −7.31) [BCa]		(−11.07; −8.41) [BCa]	
Average monthly reimbursed costs (CHF) *Follow-up phase*	1,985.87	(1,613.01; 2,358.73) [N]	2,618.68	(2,073.68; 3,163.68) [N]	1.91
	(190.24)	(1,649.32; 2,382.71) [P]	(278.07)	(2,148.84; 3,228.29) [P]	
		(1,650.26; 2,385.68) [BC]		(2,144.84; 3,224.02) [BC]	
		(1,656.47; 2,394.44) [BCa]		(2,184.60; 3,303.39) [BCa]	
Average monthly number of non-readmission days *Follow-up phase*	29.05	(28.71; 29.38) [N]	28.30	(27.71; 28.89) [N]	−2.17*
	(0.17)	(28.68; 29.34) [P]	(0.30)	(27.65; 28.83) [P]	
		(28.70; 29.36) [BC]		(27.60; 28.80) [BC]	
		(28.67; 29.33) [BCa]		(27.45; 28.76) [BCa]	

*p < 0.05, **p < 0.01, ***p < 0.001.

^a^ CRHT, Crisis Resolution Home Treatment.

^b^ CPC, Cantonal Psychiatric Clinic.

^c^ S.E., Standard Error.

^d^ CHF, Swiss Francs.

^e^ N = Normal.

^f^ P = Percentile.

^g^ BC, Bias Corrected.

^h^ BCa, Bias Corrected and accelerated.

^i^ HoNOS, health of the nations outcome scales.

Table 3 reports a difference in the adjusted total costs of CHF 3,090.86 in favour of CRHT, again not statistically significant at the 5% level (p-value = 0.079). Nevertheless, if we consider the lower risk aversion when making financial decisions instead of clinical decisions, leading to the use of 90% confidence intervals instead of 95% as suggested by McCrone et al. [51], the difference becomes significant. The adjusted difference in psychiatric symptoms between admission and discharge favouring hospitalization (+1.58 on average for CRHT) was also not significant (p-value = 0.150).

**TABLE 3:** Adjusted differences in mean costs and effectiveness measures (Mendrisio, Switzerland. 2019).

Bootstrapped clustered regression models^a^	Coefficient (bootstrap S.E.^b^)	95% confidence intervals
*Treatment phase*		
Difference in total actual treatment costs (CHF^c^)	−3,090.86	(−6,534.44; 352.72) [N^d^]
(CRHT^e^ vs. CPC^f^)	(1,756.96)	(−6,666.54; 330.91) [P^g^]
		(−6,813.23; 175.81) [BC^h^]
Adjusted R^2^ = 0.086		(−6,929.82; 165.86) [BCa^i^]
Number of observations (n) = 219		
Difference in the variation of the HoNOS^j^ total score between admission and discharge (CRHT vs. CPC)	1.58	(−0.57; 3.73) [N]
	(1.10)	(−0.74; 3.63) [P]
		(−0.46; 3.74) [BC]
Adjusted R^2^ = 0.091		(−0.45; 3.74) [BCa]
Number of observations (n) = 208		
*Follow-up phase*		
Difference in average monthly reimbursed costs in CHF (CRHT vs. CPC)	−380.18	(−998.43; 238.06) [N]
	(315.44)	(−1,017.14; 234.77) [P]
		(−1,012.87; 242.52) [BC]
Adjusted R^2^ = 0.186		(−1,017.80; 234.07) [BCa]
Number of observations (n) = 163		
Difference in average monthly number of non-readmission days (CRHT vs. CPC)	+0.74	(−0.12; 1.60) [N]
	(0.44)	(−0.07; 1.70) [P]
		(−0.07; 1.70) [BC]
Adjusted R^2^ = 0.154		(−0.02; 1.83) [BCa]
Number of observations (n) = 163		

*p < 0.05, **p < 0.01, ***p < 0.001.

^a^ All models included the following socio-demographic and clinical control variables: gender, age, Swiss citizenship, educational level, civil status (married or not), living status (living alone or not), employment status (working or not) compulsory admission (Y/N), primary diagnosis, presence of a secondary diagnosis (Y/N), number of previous hospitalizations.

The first model of the treatment phase (Difference in total actual treatment costs) also included the HoNOS score at admission.

Both models of the follow-up phase also included the total actual treatment costs.

^b^ S.E., standard error.

^c^ CHF, swiss francs.

^d^ N = normal.

^e^ CRHT, crisis resolution home treatment.

^f^ CPC, cantonal psychiatric clinic.

^g^ P = percentile.

^h^ BC, bias corrected.

^i^ BCa, Bias Corrected and accelerated.

^j^ HoNOS, health of the nations outcome scales.

**FIGURE 2:**
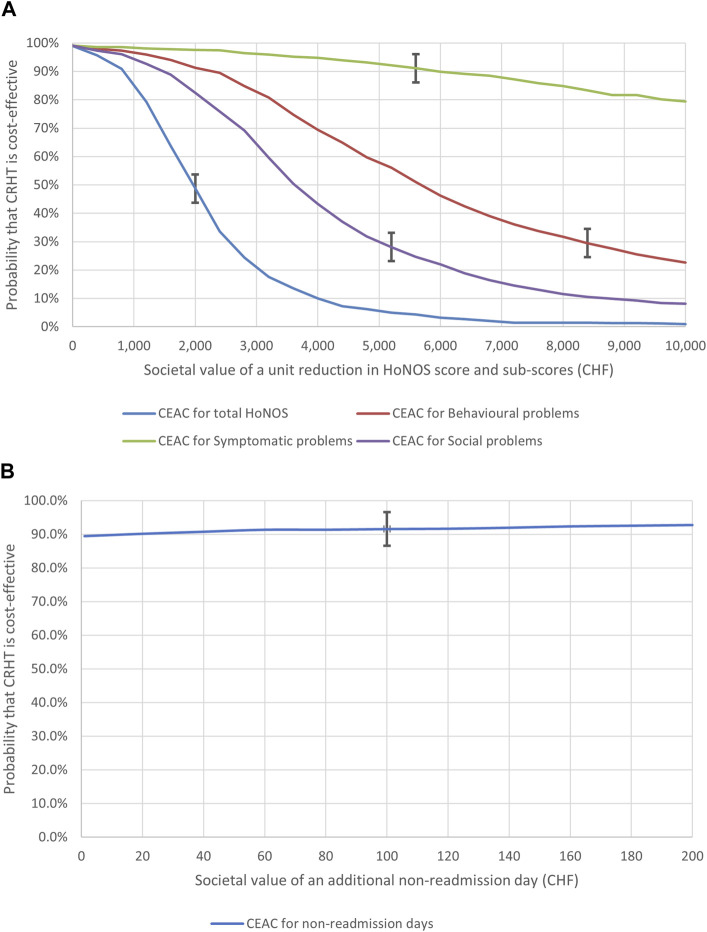
** (A, B)** Cost-Effectiveness Acceptability Curves for the treatment and follow-up phases, image have been published in the summary report cited in Flessa et al (2022). (Mendrisio, Switzerland. 2019).

The CEACs are shown in Figure 2. The treatment phase NBs were computed in both arms according to CHF 400 increments of the value of λ within a range from CHF 1 to CHF 10,000. We reported the CEAC calculated using the variation in the total HoNOS score between admission and discharge together with the CEACs computed using the variations in three HoNOS subscales (i.e.: behavioral problems, symptomatic problems, and social problems) to provide a more comprehensive evaluation. Each curve is characterized by a threshold line, which represents the monetary societal value from which the NB becomes positive. These thresholds should be used for cost-effectiveness interpretation, in the zone where the societal benefit exceeds the costs.

Overall, CRHT shows a cost-effectiveness probability falling below 50% just before the threshold, which indicates the lack of cost-effectiveness for positive NBs. The CEACs based on the HoNOS subscales indicate however very different behaviours according to the type of symptoms considered. CHRT cost-effectiveness probability resulted very low after the threshold in both CEACs based on the variation of behavioural and social symptoms (lower than 30% in both cases), while it was high (80% or more) after the threshold in the CEAC based on symptomatic problems.

### Follow-Up Phase Analysis’ Results

The CRHT unadjusted mean of average monthly costs for the follow-up phase (CHF 1,985.87) was lower than the value found for hospital treatment (CHF 2,618.68), and the difference was significant at the 10% level but not at the 5% level (p-value = 0.058). The CRHT group reported an unadjusted mean of the average number of non-readmission days (29.05) significantly higher (p-value = 0.031) than hospitalized patients (28.30), [See Table 2].

Table 3 shows adjusted mean differences in both average monthly follow-up costs (CHF -380.18) and average number of non-readmission days (+0.74) favouring CRHT over hospitalization. These differences were not statistically significant at the 5% level (p-values = 0.228 and 0.091, respectively), but using a 90% confidence level the average number of non-readmission days was significantly higher for CRHT patients.

To build the CEAC for the follow-up period, presented in Figure 2, we calculated the NBs in both arms according to CHF 20 increments of the values of λ within a range from CHF 1 to CHF 200. The CEAC indicates high cost-effectiveness probabilities of CRHT (90% or more) before and after the threshold value, with an increasing trend.

## Discussion

Our study showed that CRHT was less expensive than hospitalization both during the treatment and the follow-up periods, even if the difference was not always statistically significant. This result is perfectly in line with several previous findings [12, 14, 32–34, 38, 43, 44] and partially in line with some others reporting lower CRHT total costs but higher outpatient costs [13] and lower or higher CRHT costs depending on the medical site considered [37], but is in contrast with two other studies reporting higher CRHT costs [35, 36]. CRHT resulted cost-effective in comparison with hospitalization for the follow-up phase, but cost-effectiveness for the treatment phase depended on the types of psychiatric symptoms considered and on the theoretical value that society attributes to a one-unit increase in effectiveness (CRHT cost-effectiveness lowered as the value increased). The findings regarding the follow-up period are in line with many others reporting general cost-effectiveness of CRHT [12, 32, 34, 36–38], while the findings concerning the treatment phase are similar to others indicating that cost-effectiveness of CRHT may vary according to the value that society attributes to a one-unit increase in effectiveness [13, 14] and to the combination of CRHT and inpatient treatment received [39]. The heterogeneity of the empirical evidence presented makes it difficult to draw robust conclusions on CRHT cost-effectiveness in comparison with standard inpatient treatment.

Even if some differences between CRHT and standard inpatient treatment were not statistically significant, our study provided clear evidence in favour of CRHT for acute psychiatric crisis management in Southern Switzerland. CHRT was both cheaper and comparable to hospitalization in terms of clinical effectiveness during the treatment phase, and highly cost-effective in the follow-up phase. From the practical point of view, CRHT should be offered to all patients with an acute psychiatric crisis eligible for the service to provide a more efficient allocation of resources. However, logistic constraints in case of high demand for the CRHT service may hinder this possibility. In this sense, it is of fundamental importance to assess in deeper details the conditions in which CRHT works best. The great variability of CRHT cost-effectiveness according to the type of psychiatric symptoms found during the treatment phase, together with the variability of CRHT cost-effectiveness related to the combination of CRHT and inpatient treatment received by patients found in a previous study [39], allowed highlighting the fact that CRHT cost-effectiveness may depend on specific patients’ profiles and treatment patterns. Similar results were found for CRHT clinical effectiveness evaluation, which led to the first research contributions exploring the specific characteristics that make patients better suited for CRHT [52] and that are related to relapses after CRHT [53]. Accordingly, research efforts exploring the specific patients’ characteristics related to CRHT cost-effectiveness are needed, to obtain information useful for an even more efficient resources’ allocation and, if necessary, for patients’ prioritization in case of an excess demand for the CRHT service Important to note that a generalization of access to CRHT for all patients in Southern Switzerland was included in the cantonal socio-psychiatric planning dispatch 2022–2025, and the service has since been opened for residents in the southern part as well.

The positive NB threshold for the interpretation of the CEACs, to our knowledge firstly used in this type of studies, has the advantage of being independent from subjective evaluations, and could represent a useful benchmark in a setting where the “right” monetary societal value for treatment effectiveness is difficult to establish.

Our study is limited by the lack of randomization (however at least approximately overcome using the quasi-experimental design) and the relatively limited sample size, which may increase the probability of type II errors (i.e. wrong assessment of the lack of statistical significance for differences between groups). Moreover, despite having used a robust methodological approach with the inclusion of important socio-demographic and clinical control variables in the regression models, we cannot exclude the lack of relevant variables. Also, different cost data sources for the treatment and follow-up periods may prevent from the direct comparability of the results. Finally, the single-centre nature of the study does not allow for external validity and generalizability of the findings to other settings.

In conclusion, the results of our study showed that in general CRHT can be a cost-effective alternative to standard inpatient treatment for the management of acute psychiatric crises, but this finding is not homogeneously supported in the literature because of the limited number of studies combined with the significant heterogeneity in the study settings. We also found that cost-effectiveness of CRHT varies based on the type of psychiatric symptoms considered, which is in line with the results of another study according to which CRHT cost-effectiveness is related to combination of CRHT and inpatient treatment received. These findings suggested that CRHT cost-effectiveness may be related to specific patients’ profiles and treatment patterns. Our results confirmed the strong need for further detailed research aimed at exploring the specific conditions and patients’ characteristics associated with CRHT cost-effectiveness.

## References

[1] OECD/European Union. Promoting Mental Health in Europe: Why and How? In: Health at a Glance: Europe 2018: State of Health in the EU Cycle. Paris: OECD Publishing (2018). 10.1787/health_glance_eur-2018-en

[2] Seabury SA Axeen S Pauley G Tysinger B Schlosser D Hernandez JB Measuring the Lifetime Costs of Serious Mental Illness and the Mitigating Effects of Educational Attainment. Health Aff (2019) 38(4):652–9. 10.1377/hlthaff.2018.05246 PMC6597007 30933598

[3] Thornicroft G Tansella M. The Balanced Care Model for Global Mental Health. Psychol Med (2013) 43(4):849–63. 10.1017/S0033291712001420 22785067

[4] Thornicroft G Tansella M. Components of a Modern Mental Health Service: A Pragmatic Balance of Community and Hospital Care: Overview of Systematic Evidence. The Br J Psychiatry (2004) 185(4):283–90. 10.1192/bjp.185.4.283 15458987

[5] Thornicroft G Tansella M. Balancing Community-Based and Hospital-Based Mental Health Care. World Psychiatry (2002) 1(2):84–90. 10.1097/nmd.0000000000000151 16946858 PMC1489876

[6] Smyth MG Pelosi AJ Hoult J Jackson GA. The Home Treatment Enigma, Home Treatment—Enigmas and Fantasies. BMJ: Br Med J (2000) 320(7230):305–9. 10.1136/bmj.320.7230.305 10650032 PMC1117491

[7] Johnson S Needle J Bindman JP Thornicroft G. Crisis Resolution and Home Treatment in Mental Health. New York: Cambridge University Press (2008).

[8] Gaebel W Becker T Janssen B Munk-Jorgensen P Musalek M Rössler W EPA Guidance on the Quality of Mental Health Services. Eur Psychiatry (2012) 27(2):87–113. 10.1016/j.eurpsy.2011.12.001 22264656

[9] Murphy SM Irving CB Adams CE Waqar M. Crisis Intervention for People with Severe Mental Illnesses. Cochrane Database Syst Rev (2015) 2015(12):CD001087. 10.1002/14651858.CD001087.pub5 26633650 PMC7052814

[10] Burns T Knapp M Catty J Healey A Henderson J Watt H Home Treatment for Mental Health Problems: A Systematic Review. Health Technology Assess (2001) 5(15):1–139. 10.3310/hta5150 11532236

[11] Soldini E Alippi M Zufferey MC Lisi A Lucchini M Albanese E Effectiveness of Crisis Resolution Home Treatment for the Management of Acute Psychiatric Crises in Southern Switzerland: A Natural Experiment Based on Geography. BMC Psychiatry (2022) 22(1):405. 10.1186/s12888-022-04020-z 35715789 PMC9204869

[12] Fenton WS Hoch JS Herrell JM Mosher L Dixon L. Cost and Cost-Effectiveness of Hospital vs Residential Crisis Care for Patients Who Have Serious Mental Illness. Arch Gen Psychiatry (2002) 59(4):357–64. 10.1001/archpsyc.59.4.357 11926936

[13] McCrone P Johnson S Nolan F Pilling S Sandor A Hoult J Economic Evaluation of a Crisis Resolution Service: A Randomised Controlled Trial. Epidemiol Psychiatr Sci (2009) 18(1):54–8. 10.1017/s1121189x00001469 19378700

[14] Kilian R Becker T Frasch K. Effectiveness and Cost-Effectiveness of Home Treatment Compared with Inpatient Care for Patients with Acute Mental Disorders in a Rural Catchment Area in Germany. Neurology. Psychiatry Brain Res (2016) 22(2):81–6. 10.1016/j.npbr.2016.01.005

[15] Gournay K Brooking J. The Community Psychiatric Nurse in Primary Care: An Economic Analysis. J Adv Nurs (1995) 22(4):769–78. 10.1046/j.1365-2648.1995.22040769.x 8708198

[16] McCrone P Beecham J Knapp M. Community Psychiatric Nurse Teams: Cost-Effectiveness of Intensive Support versus Generic Care. The Br J Psychiatry (1994) 165(2):218–21. 10.1192/bjp.165.2.218 7953035

[17] Mangen SP Paykel ES Griffith JH Burchell A Mancini P. Cost-effectiveness of Community Psychiatric Nurse or Out-Patient Psychiatrist Care of Neurotic Patients. Psychol Med (1983) 13(2):407–16. 10.1017/s0033291700051047 6410436

[18] McCrone P Thornicroft G Phelan M Holloway F Wykes T Johnson S. Utilisation and Costs of Community Mental Health Services: PRiSM Psychosis Study 5. The Br J Psychiatry (1998) 173(5):391–8. 10.1192/bjp.173.5.391 9926055

[19] Tyrer P Evans K Gandhi N Lamont A Harrison-Read P Johnson T. Randomised Controlled Trial of Two Models of Care for Discharged Psychiatric Patients. BMJ: Br Med J (1998) 316(7125):106–9. 10.1136/bmj.316.7125.106 9462315 PMC2665389

[20] Byford S Fiander M Torgerson DJ Barber JA Thompson SG Burns T Cost-effectiveness of Intensive V. Standard Case Management for Severe Psychotic Illness: UK700 Case Management Trial. The Br J Psychiatry (2000) 176(6):537–43. 10.1192/bjp.176.6.537 10974959

[21] Bond GR Miller LD Krumwied RD Ward RS. Assertive Case Management in Three CMHCs: A Controlled Study. Psychiatr Serv (1988) 39(4):411–8. 10.1176/ps.39.4.411 2836295

[22] Chandler D Meisel J Hu TW McGowen M Madison K. Client Outcomes in a Three-Year Controlled Study of an Integrated Service Agency Model. Psychiatr Serv (1996) 47(12):1337–43. 10.1176/ps.47.12.1337 9117472

[23] Clark RE Teague GB Ricketts SK Bush PW Xie H McGuire TG Cost-effectiveness of Assertive Community Treatment versus Standard Case Management for Persons with Co-occurring Severe Mental Illness and Substance Use Disorders. Health Serv Res (1998) 33(5 Pt 1):1285–308. 9865221 PMC1070317

[24] Essock SM Frisman LK Kontos NJ. Cost‐effectiveness of Assertive Community Treatment Teams. Am J Orthopsychiatry (1998) 68(2):179–90. 10.1037/h0080328 9589757

[25] Lehman AF Dixon L Hoch JS DeForge B Kernan E Frank R. Cost-effectiveness of Assertive Community Treatment for Homeless Persons with Severe Mental Illness. The Br J Psychiatry (1999) 174(4):346–52. 10.1192/bjp.174.4.346 10533554

[26] McGurrin MC Worley N. Evaluation of Intensive Case Management for Seriously and Persistently Mentally Ill Persons. J Case Management (1993) 2(2):59–65. 8130745

[27] Wolff N Helminiak TW Morse GA Calsyn RJ Klinkenberg WD Trusty ML. Cost-effectiveness Evaluation of Three Approaches to Case Management for Homeless Mentally Ill Clients. Am J Psychiatry (1997) 154(3):341–8. 10.1176/ajp.154.3.341 9054781

[28] Stein LI Test MA Marx AJ. Alternative to the Hospital: A Controlled Study. The Am J Psychiatry (1975) 132(5):517–22. 10.1176/ajp.132.5.517 164129

[29] Fenton FR Tessier L Struening EL. A Comparative Trial of Home and Hospital Psychiatric Care: One-Year Follow-Up. Arch Gen Psychiatry (1979) 36(10):1073–9. 10.1001/archpsyc.1979.01780100043003 475542

[30] Muijen M Marks IM Connolly J Audini B McNamee G. The Daily Living Programme: Preliminary Comparison of Community versus Hospital-Based Treatment for the Seriously Mentally Ill Facing Emergency Admission. The Br J Psychiatry (1992) 160(3):379–84. 10.1192/bjp.160.3.379 1562865

[31] Hoult J Reynolds I Charbonneau-Powis M Weekes P Briggs J. Psychiatric Hospital versus Community Treatment: The Results of a Randomised Trial. Aust New Zealand J Psychiatry (1983) 17(2):160–7. 10.3109/00048678309160000 6578788

[32] Knapp M Beecham J Koutsogeorgopoulou V Hallam A Fenyo A Marks IM Service Use and Costs of Home-Based versus Hospital-Based Care for People with Serious Mental Illness. The Br J Psychiatry (1994) 165(2):195–203. 10.1192/bjp.165.2.195 7953032

[33] Knapp MR Marks IM Wolstenholme J Beecham JK Astin J Audini B Home-Based versus Hospital-Based Care for Serious Mental Illness: Controlled Cost-Effectiveness Study over Four Years. The Br J Psychiatry (1998) 172(6):506–12. 10.1192/bjp.172.6.506 9828991

[34] Merson S Tyrer P Carlen D Johnson T. The Cost of Treatment of Psychiatric Emergencies: A Comparison of Hospital and Community Services. Psychol Med (1996) 26(4):727–34. 10.1017/s0033291700037740 8817707

[35] Gater R Goldberg D Jackson G Jennett N Lowson K Ratcliffe J The Care of Patients with Chronic Schizophrenia: A Comparison between Two Services. Psychol Med (1997) 27(6):1325–36. 10.1017/s0033291797005631 9403904

[36] Weisbrod BA Test MA Stein LI. Alternative to Mental Hospital Treatment: II. Economic Benefit-Cost Analysis. Arch Gen Psychiatry (1980) 37(4):400–5. 10.1001/archpsyc.1980.01780170042004 6767462

[37] Rosenheck RA Neale MS. Cost-effectiveness of Intensive Psychiatric Community Care for High Users of Inpatient Services. Arch Gen Psychiatry (1998) 55(5):459–66. 10.1001/archpsyc.55.5.459 9596049

[38] Fenton FR Tessier L Contandriopoulos AP Nguyen H Struening EL. A Comparative Trial of Home and Hospital Psychiatric Treatment: Financial Costs. The Can J Psychiatry (1982) 27(3):177–87. 10.1177/070674378202700301 6807524

[39] Coates DB Kendall LM MaCurdy EA Goodacre RH. Evaluating Hospital and Home Treatment for Psychiatric Patients. Canada's Ment Health (1976) 24(1):28–33. 828531

[40] Morandi S Silva B Golay P Bonsack C. Intensive Case Management for Addiction to Promote Engagement with Care of People with Severe Mental and Substance Use Disorders: An Observational Study. Substance Abuse Treat Prev Policy (2017) 12:26–0. 10.1186/s13011-017-0111-8 PMC5445418 28545572

[41] Bonsack C Golay P Gibellini Manetti S Gebel S Ferrari P Besse C Linking Primary and Secondary Care after Psychiatric Hospitalization: Comparison between Transitional Case Management Setting and Routine Care for Common Mental Disorders. Front Psychiatry (2016) 7:96. 10.3389/fpsyt.2016.00096 27313547 PMC4889580

[42] Bonsack C Adam L Haefliger T Besson J Conus P. Difficult-to-engage Patients: A Specific Target for Time-Limited Assertive Outreach in a Swiss Setting. The Can J Psychiatry (2005) 50(13):845–50. 10.1177/070674370505001307 16483119

[43] Kraan K. Integrated Home Treatment in Acute Episodes of Mental Illness. Results from a Pilot Project in Lucerne. InEuropean Congress Social Psychiatry (2012) 4:4–6.

[44] Stulz N Wyder L Maeck L Hilpert M Lerzer H Zander E Home Treatment for Acute Mental Healthcare: Randomised Controlled Trial. The Br J Psychiatry (2020) 216(6):323–30. 10.1192/bjp.2019.31 30864532

[45] Mötteli S Schori D Schmidt H Seifritz E Jäger M. Utilization and Effectiveness of Home Treatment for People with Acute Severe Mental Illness: A Propensity-Score Matching Analysis of 19 Months of Observation. Front Psychiatry (2018) 9:495. 10.3389/fpsyt.2018.00495 30364109 PMC6191514

[46] Levati S Mellacqua Z Caiata-Zufferey M Soldini E Albanese E Alippi M Home Treatment for Acute Mental Health Care: Protocol for the Financial Outputs, Risks, Efficacy, Satisfaction Index and Gatekeeping of Home Treatment (FORESIGHT) Study. JMIR Res Protoc (2021) 10(11):e28191. 10.2196/28191 34751660 PMC8663595

[47] Flessa S Gächter T Gerfin M Plaza de Laifer C Siqeca F Yip O. Synthesis Working Paper: Cost and Reimbursement. Switzerland: Bern (2022). Available online at: https://www.nfp74.ch/en/WW3T81gCZ6vTbLpX/page/findings (Accessed December 16, 2024).

[48] Keele L Titiunik R. Natural Experiments Based on Geography. Polit Sci Res Methods (2016) 4(1):65–95. 10.1017/psrm.2015.4

[49] Wing JK Beevor AS Curtis RH Park SG Hadden J Burns A. Health of the Nation Outcome Scales (HoNOS): Research and Development. The Br J Psychiatry (1998) 172(1):11–8. 10.1192/bjp.172.1.11 9534825

[50] Johnson S Nolan F Pilling S Sandor A Hoult J McKenzie N Randomised Controlled Trial of Acute Mental Health Care by a Crisis Resolution Team: The North Islington Crisis Study. Bmj (2005) 331(7517):599. 10.1136/bmj.38519.678148.8F 16103032 PMC1215550

[51] McCrone P Knapp M Proudfoot J Ryden C Cavanagh K Shapiro DA Cost-effectiveness of Computerised Cognitive-Behavioural Therapy for Anxiety and Depression in Primary Care: Randomised Controlled Trial. The Br J Psychiatry (2004) 185(1):55–62. 10.1192/bjp.185.1.55 15231556

[52] Mötteli S Jäger M Hepp U Wyder L Vetter S Seifritz E Home Treatment for Acute Mental Healthcare: Who Benefits Most? Community Ment Health J (2021) 57:828–35. 10.1007/s10597-020-00618-3 32279118

[53] Werbeloff N Chang CK Broadbent M Hayes JF Stewart R Osborn DP. Admission to Acute Mental Health Services after Contact with Crisis Resolution and Home Treatment Teams: An Investigation in Two Large Mental Health-Care Providers. The Lancet Psychiatry (2017) 4(1):49–56. 10.1016/S2215-0366(16)30416-3 27979719

